# Common Risk Factors for Urinary House Soiling (Periuria) in Cats and Its Differentiation: The Sensitivity and Specificity of Common Diagnostic Signs

**DOI:** 10.3389/fvets.2018.00108

**Published:** 2018-05-28

**Authors:** Ana Maria Barcelos, Kevin McPeake, Nadja Affenzeller, Daniel Simon Mills

**Affiliations:** ^1^Animal Behaviour Cognition and Welfare Group, School of Life Sciences, University of Lincoln, Lincoln, United Kingdom; ^2^Department for Companion Animals and Horses, Clinical Unit of Internal Medicine Small Animals, University of Veterinary Medicine Vienna, Vienna, Austria

**Keywords:** cat, house soiling, inappropriate urination, latrine, marking, spraying, toileting

## Abstract

Urinary house soiling (periuria) in the home is a common but serious behaviour problem in cats. Although many specific risk factors and triggers have been postulated, their importance is largely unknown. This study assessed: (1) the significance of purported risk factors for periuria as well as specifically marking and latrine behaviour in the home; (2) the specificity and sensitivity of signs commonly used to differentiate latrine and marking behaviour. Owner responses to an internet survey (*n* = 245) were classified into three groups: control, marking and latrine behaviour, along with 41 potential risk factors and 15 predictors used to diagnose marking and latrine problems. Univariate statistical analyses and non-parametric tests of association were used to determine simple associations. In addition the sensitivity and specificity of four cardinal signs (posture to urinate, attempt to cover soiled area, surface chosen and volume of urine deposited) were calculated. Significant potential risk factors were: age (marking cats were older than the other two groups); multi-cat household (increased risk of marking and latrine behaviours); free outside access and cat flaps in the house (higher frequency of marking); outside access in general (lower prevalence of latrine behaviour); defecation outside the litter box (higher frequency of latrine behaviour); a heavy dependence by the cat on its owner (lower frequency of latrine behaviour) and a relaxed personality (lower risk of marking behaviour). Litterbox attributes and disease related factors were not significant. Individual cardinal signs were generally not good predictors of diagnosis. This study challenges the poor quality of evidence that has underpinned some of the hypotheses concerning the causes of periuria in cats. The results, in particular, highlight the general importance of the social environment, with the presence of other cats in the household, the cat-owner bond and personality related factors, alongside factors like the use of a cat flap which might also alter the social environment, all implicated as significant risk factors. While the physical environment may be important in specific cases, it seems this is less important as a general risk factor. The findings quantify the risk of misdiagnosis if a single sign is considered sufficient for diagnosis.

## Introduction

House soiling, also sometimes referred to as inappropriate or undesirable elimination (1–4), is one of the most common behaviour problems reported in cats in many countries (1, 3, 5–11) and is one of the main reasons for cat relinquishment (12, 13). The term house soiling includes both urine and faeces being deposited in the home outside of any litter box regardless of whether this is a latrine-related issue (3, 7, 14), with urine-related complaints the more prevalent of the two (3, 7, 11).

House soiling with urine, which may also be referred to less ambiguously as periuria (14), is traditionally considered in two categories: marking [sometimes called spraying (2, 15), although the characteristic spray posture may not always be present (5, 14, 16)] and latrine related behaviour, which is also frequently referred to as inappropriate urination (17) or inappropriate toileting (4, 14, 18, 19), although the latter term clearly includes defecation as well (14, 20). In this study, the general deposition of urine outside the litter box, regardless of the specific diagnosis, will be referred to as urinary house soiling or periuria.

Urine marks are believed to be one of the basic forms of chemical signal (alongside faeces and secretions from skin glands) (5, 21) widely used in communication by cats (4, 5, 14, 16, 18, 22). Although marking using other media is possible (5), the term “marking”, will be used in this report to refer to urine marking only, unless specifically indicated otherwise. Likewise, latrine related problems may include problems involving faeces (14), but in the current context, we will use the term latrine behaviour to refer to non-marking urination, associated with the decision to use a new toilet area in the home (16–18) to evacuate the bladder of its stored contents (4, 5, 14, 16).

Veterinarians are viewed by many owners as an important source of potential help with house soiling problems, with 74% of cat owners reported in one study to seek help from their veterinarians for urine marking problems (23). It is therefore important to have simple means to readily differentiate or screen these problems, since most cases may be initially treated in general practice, where consultation time is often very limited. It is frequently suggested that certain postural and behavioural characteristics associated with the behaviour may be useful in this regard (7, 24); however, it seems that these features may not be widely appreciated by general practitioners, since in one study (23), almost a third of veterinarians could not correctly describe some of them. Besides the lack of a complete understanding of house soiling by veterinarians, there is the wider issue that the scientific evidence for the traditional clinical signs extensively used by behaviourists to diagnose the cause of periuria is weak. Marking, for example, is frequently associated with a standing posture, with an upright and quivering tail, a small amount of urine, which is deposited on vertical surfaces in certain specific contexts (territorial, sexual, competitive) in areas of social importance (next to doors or windows) (1, 4, 5, 9, 21, 23, 25). By contrast, latrine related behaviour is often described as involving a squatting posture, a large volume of urine on horizontal surface and hidden or more secluded locations, and being followed by digging and covering behaviour (1, 4, 5, 9, 25). There is therefore a need to examine true value of these signs in terms of their sensitivity (i.e., proportion of cats with a given form of periuria who are correctly identified by each of these signs) and specificity (i.e., proportion of cats without a given form of periuria who are correctly identified by the absence of each of these signs).

Many risk factors have also been postulated for general urinary house soiling, including anxiety, the absence of the owner, being in a multi-cat household, and significant social or environmental change (e.g., a new animal or person in the home) as well as learned negative associations with the litter box and certain medical conditions, including idiopathic cystitis, pain and chronic kidney disease (1, 15, 21, 26–28). Specific risk factors and triggers for the different forms of periuria have also been suggested and widely accepted without much apparent questioning or evidence. For example in the case of marking, this includes a genetic predisposition, excitement and/or stress, not being neutered, being in oestrus, being male, inappropriate attempts at punishment, specific inter-cat situations (such as aggressive encounters, territorial disputes or simply the sight or noise of an outdoor cat), novel scent on house objects, perturbation of familiar marking, and certain specific environmental changes, such as new furniture (5, 9, 15, 18, 21, 22, 25, 27, 29, 30). For latrine related behaviour outside of the litter box suggested specific risk factors and triggers include a range attributes of the latrine such as those relating to the quality of the litter (individual preference, a new depth of litter, granule size, new litter, reduced cleaning regime) or litter box (covered versus uncovered, location, quantity, size, style, use of box liners or electric self-cleaning box), as well as a wide range of medical conditions, including age-related conditions e.g., hyperthyroidism, arthritis and cognitive dysfunction/senility as well as dysuria and conditions associated with polyuria such as diabetes or renal failure, in addition to neurologic and sensory impairment especially in relation to olfaction and vision, as well as more general weakness (1, 2, 4, 5, 9, 15, 16, 18, 21, 25, 27, 29, 31, 32).

Many of these potential associations, if they do genuinely exist, are quite non-specific and the actual trigger may be one of several related factors. Without good scientific data, ideological associations may inadvertently be believed to be genuine risk factors. For example, Ellis et al. (20) investigated the influence of previous litter box use on its reuse and found through careful experimental manipulation that it was not the odor of another cat's urine or faeces in the litter that was avoided by cats (obvious association for a macrosmatic species like the cat), but the physical/visual presence of a wet patch or object looking like a faecal deposit which mattered. This work highlights the importance of original research which challenges conventional wisdom on this topic and the danger of uncritically accepting the opinion of others without good data. It is perhaps surprising that until now (see Tables 1–3) there has been no synthesis of the available data on risk factors, and these Tables highlight its generally low quality from an evidence-based perspective. Very few of the postulated risk factors described above are substantiated by any form of data. Even for those with data, the effect may be inconsistent and rarely has the strength of the association been quantified; therefore, in Tables 1–3, we report, where available, the original authors’ quantification of effect and, where possible, our calculation of this from the data published. This shows that, for example, although multi-cat households are widely believed to be at higher risk of marking behaviour problems, Olm and Houpt’s (1) results were non-significant for this factor, whereas Pryor et al. (17) found it to be significant, but only with a one sided statistical test, that would have been non-significant if the more conventional 2-sided test was used. Likewise, Herron (4) suggested that covered litter boxes could be a cause of latrine related behaviour problems in cats, but in the only known study investigating this, Grigg et al. (33) subsequently found no significant difference between the use of uncovered and covered litter boxes. It is therefore clear that the evidence relating to potential associations with and causes of periuria in cats is weak and in need of scientific data to support or refute it.

**Table 1:** Risk factors for a **urinary marking problem** in the home and associated data from a review of the literature.

**Risk factor**	**Study reference and population characteristics**	**Metric:OR (CI)**	**Significance**	**Analysis used to extract risk factor**
**Gender / neuter status**				
Male	(34)(*n* = 134 male neutered, 152 female neutered cats)	**OR = 2.30(1.29–4.11)**	***p* = 0.004**	Case series prevalence comparison vs female neutered
	(1)(*n* = 19 marking, 30 latrine behaviour cats)	**OR = 4.83(1.37–17.09)**	***p* = 0.012**	Case series prevalence comparison (male versus female)
	(17) (*n* = 47 marking cats recruited for intervention study)		*p* < 0.001(1-sided test)	Enrolment characteristics comparison with population of pet cats in California
Castration of males	(35)(*n* = 23 marking cats subject to castration)	**20/23(87%) reduce**		Case series comparison (pre and post castration)
Early-age (<5.5 mo) castration in males	(36)	OR = 0.79 (0.64–0.97)	*p* = 0.002	Retrospective study, OR for 1 mo reduction in neutering age
**Outdoor access**	(17)	12/47 (26%)		Owner reported significance to problem
**Reduced cleaning regime (litter box and marks)**	(17)		*p* < 0.001	Case series, within subjects comparison based on intervention effects
**Medical-related conditions**				
Urinary abnormalities	(37) (*n* = 34 marking cats that use vertical surfaces only)	13/34 (38%)		Case series, within subjects comparison
	(38) (*n* = 58 neutered marking cats, *n* = 39 controls)		*p* > 0.05	Case-control, comparison based on urinalysis results
Lower urinary tract disease	(1)	**OR = 0.71(0.20–2.55)**	***p* = 0.604**	Case series prevalence comparison with latrine cases
**Social-related**				
Inter-cat antagonism	(17)	49% outside the home 28% in the home		Owner reported significance to problem
Multi-cat household	(1)	**OR = 2.67(0.63–11.35)**	***p* = 0.175**	Case series prevalence cf latrine cases
	(17)		*p* = 0.032 (1-tail test)	Cross-sectional comparison based on enrolment characteristics

OR (CI) = Odds ratio and 95% CI. Results in bold have been calculated by the current authors from the available data, rather than authors of the original study. Blank cells indicate data not available.

**Table 2:** Risk factors for **urinary latrine problem** in the home and associated data from a review of the literature.

**Risk factors**	**Study reference and population characteristics**	**Metric:OR (CI)**	**Significance**	**Analysis used to extract risk factor**
**Gender/neuter status**				
Age of neutering	(39) (*n* = 126, 21 urinary latrine and 105 control cats at 6–13 weeks of age)		*p* > 0.05	Cohort study, influence of the age of neutering on house soiling post adoption
Intact female kittens	(39)	**OR = 5.58(1.10–28.20)**	*p* < 0.05	Case-control comparison with neutered females kittens
**Defecation outside the litter box**	(40) (*n* = 60 latrine cats)	25/56 (45%)		Case series prevalence (latrine only cases = 56/60)
	(1) (*n* = 30 latrine cats)	**11/30(36.7%)**		Case series prevalence (cf 0/19 marking cats)
	(39)	**OR = 10.33(3.61–29.61)**	*p* < 0.001	Case-control comparison
**Medical-related conditions**				
Lower urinary tract disease in the past	(1)	**10/30 (33%) OR = 1.40(0.39–5.00)**	***p* = 0.604**	Case series prevalence comparison with marking cats
**Social related**				
Aggression to family members	(39) (*n* = 126 kittens, 27 urinary/fecal latrine problem, 99 controls)	11/27 (41%)** OR = 2.406(0.98–5.93)**	*p* > 0.05	Case-control comparison cases include cats with fecal latrine problem, controls = no latrine problem
Multi-cat household	(1)	**OR = 0.38(0.09–1.60)**	***p* = 0.175**	Case series prevalence comparison with marking cats
	(39) (*n* = 21 latrine kittens)		*p* > 0.05	Case control comparison with cats with no latrine issues

OR (CI) = Odds ratio and 95% CI. Results in bold have been calculated by the current authors from the available data, rather than authors of the original study. Blank cells indicate data not available.

**Table 3:** Risk factors for **urinary house soiling (periuria)** in the home and associated data from a review of the literature.

**Risk factors**	**Study reference and population characteristics**	**Metric: OR (CI)**	**Significance**	**Analysis used to extract risk factor**
**Medical-related**				
Urinary tract disease in the past	(2) (*n* = 100 house soiling cats *n* = 44 non-house soiling controls)	**OR = 3.88(1.50–10.05)**	*p* < 0.05	Case-control comparison
**Breed**				
Bengal	(41) (*n* = 574 cats)		*p* = 0.001	Cross-sectional survey
Birman	(41)		*p* = 0.02	Cross-sectional survey
Persian	(3) (*n* = 336 behaviour problem cats, of which 131 house soiling. 189 controls)		*P* < 0.01	Case- control comparison
	(11) (*n* = 1556 cats with behaviour problems, 470 cats with urinary latrine behaviour with or without faeces as well)	**OR = 2.04(1.54–2.70)78/244(32%)**	*p* < 0.001*	Cross-sectional survey
Siamese type	(11)	**OR = 1.64(1.09–2.45)43/106 (40.6%)**	*p* = 0.016*	Cross-sectional study
**Emotional conditions**				
Separation anxiety (SA)	(42) (*n* = 716 behaviour problems cats, 136 SA cases)	**96/136 (70.6%)**“Five times more likely in SA cats”	*p* < 0.001	Case-control comparison
**Litter-box related**				
Absence of covering both urine and stool in the litter box	(2)	**OR = 3.65(1.72–7.75)**	*p* < 0.01	Case-control comparison
Scented litter	(2)	**OR = 6.37(2.86–14.20)**	*p* < 0.01	Case-control comparison
**Less time spent digging in the litter box**	(31)(*n* = 40 cats, 20 with elimination problems)		*p* = 0.002	Case-control comparison

OR (CI) = Odds ratio and 95% CI. Results in bold have been calculated by the current authors from the available data, rather than authors of the original study. Blank cells indicate data not available.

* Calculation here based on population of cats that deposit both urine and faeces outside the litter box as well as cats that only deposited urine outside the box, unlike the original study.

Given the clear significance of the problem of urinary house soiling in cats and its potentially fatal consequences, it is perhaps surprising that our understanding of the problem seems to be largely based on such weak evidence, with the little data that is available often being equivocal. To date there does not appear to be any form of epidemiological study designed to target this issue published in the peer reviewed literature. Univariate analyses of association are preferred in this sort of examination of perceptions, since they reveal strong general associations with relatively small samples and allow the exploration of the validity of many individual perceived associations; by contrast multivariate modeling requires very large samples when there are many potential predictors of the dependent variable, and individual relationships may be lost if they correlate with a better predictor. Therefore, the aim of this study was to undertake a survey that would allow us to initially quantify the significance of a range of potential risk factors associated with marking, latrine and urinary house soiling in general; we also aimed to determine the sensitivity (probability of a positive diagnosis) and specificity (probability of absence of the sign correctly excluding the diagnosis) of specific signs traditionally related to the different forms of the problem.

## Material and Methods

### Questionnaire

A Brazilian Portuguese and UK English questionnaire (See  Appendix I Data Sheet S1 for English version) was designed for cat owners in order to gather demographic data about the cat (5 items relating to the owner’s country, cat’s age, breed, gender and reproductive status), and information on purported potential risk factors for both marking and latrine behaviour in cats and observed features associated with the act of periuria (based on the literature and clinical experience). These later items focused on the cat's posture during urination, behaviour after the urination, the surface chosen, an estimate of the amount of urine eliminated, substrates and areas chosen. The items were in both an open format (34 items, e.g., cleaning products used) and closed format (26 items, e.g., if the cat has access to the outside yes/no). The survey was made available online only, and was shared via social media, from April to June 2016, making it clear that we were interested in the elimination behaviour of cats, but not specifically house soiling problems.

If respondents had observed urine outside of the litter box in the home they were directed to complete the whole survey in relation to that cat, whereas those who had not were directed to complete only 34 further items (from question 22 onwards). Items answered by all cat owners (1, 22–55), apart from the second one, were assessed as potential risk factors, as they could potentially be used for statistical comparison among the three groups studied (non-house soiling cats, cats with marking and cats with latrine behaviour). On the other hand, the items only answered by owners of house soiling cats (3–21) are referred to as predictors of periuria, as they could be used just to compare two groups of cats with periuria (cats with marking behaviour and with latrine behaviour).

### Exclusion Criteria

No pre-enrolment exclusion criteria were applied in relation to owners or their cats. We used a convenience sample (43) based on internet accessibility and snowball sampling through social media. Subsequent exclusion criteria were applied to those that did not answer the second item which asked whether or not they had ever observed urinary house soiling by their cat and/or did not complete more than 60% of the questionnaire; in both cases none of the data relating to these respondents has been used in this study.

### Definition of Groups for Comparison

Definition of non-periuric cats was by owner self-declaration, through their response to the item asking if they had ever observed urine marking or toileting behaviour in the home, cats with periuria were identified by a positive response to this item and those relating to current urinary house soiling activity. The specific determination of whether the cat was engaged in marking and/or latrine behaviour was from initial evaluation of the whole questionnaire by the first author, with subsequent confirmation by a professionally recognised veterinary behaviour specialist (DM). As specialists, the veterinarians not only assessed answers related to traditional indicators of periuria (the posture used to urinate, the surface chosen, the volume of urine deposited and the presence or absence of attempt to cover the area), but also all the general information from the questionnaire such as the cat’s gender, its reproductive status, location of the urine (near the door, on clothes, near the litter box, etc.), antecedents of the behaviour (e.g., the sight of another cat outside), number of cats in the household, frequency the owner cleaned the litter boxes, number of litter boxes, medical history. Subsequently, when the responses fulfilled predominantly the characteristics of either marking or latrine behaviour and were in accordance with the clinical judgement, the diagnosis was finally made. In six cases, where there was uncertainty or disagreements, two independent veterinary behaviour medicine residents (NA and KM) were asked to evaluate the questionnaires and a consensus was established by the team.

### Definition of Potential Risk Factors Groups

Overall, 41 potential risk factors were evaluated and divided into the following categories (note some items included several risk factors): individual demographics (question 1, relating to age, breed and gender including neuter status of the cat), household (questions 22, 37, 38, 40, 50), litter box attributes (questions 23–27, 34) and cat's individual behavioural and medical characteristics (questions 28, 35, 36, 41–49, 51–54). One item, relating to depth of litter/litter tray was excluded from analysis due to the subsequent identification of a translation error in this item between the two versions.

### Predictors Used for Marking and Latrine Differentiation

Despite there being no accepted algorithm for making a diagnosis, weight is usually given to a range of house soiling predictors described in classic texts, such as posture, surface chosen (vertical or horizontal), location in the house, amount of urine deposited, substrate preference (present or absent) and complete or partial reduction of litter box use (14, 29), with no single feature sufficient for a diagnosis. Thus it is useful to determine how consistently each of these signs might be being used in this process. Nineteen items (items 3 to 21) were therefore examined in this regard to determine their association with either marking or latrine behaviour. Those found to be significant were examined further to determine both their sensitivity and specificity as this indicates their diagnostic value and the level of error associated with using them as diagnostic criteria.

### Statistical Analysis

Only those items with more than three-quarters usable answers were considered for statistical analysis. After initial assessment of the population characteristics of cats relating to the language of questionnaire completed, data from the two surveys were pooled in order to examine the significance of possible risk factors. Since our aim was to determine whether or not certain associations were potentially statistically significant, we did not wish to exclude correlating factors, and so univariate analyses were undertaken. Although we undertook multiple testing, we used a significance threshold of 0.05, since the impact of a type 1 statistical error was considered less important than a type 2 error, because we were mainly focused on assessing the evidence for the risk factors postulated in the literature rather than the identification of new risk factors. Data were analysed using non-parametric tests of association (Chi-square tests, Mantel-Haenszel Common Odds Ratio Estimate and Spearman’s rank correlation coefficient) and difference (Kruskal-Wallis tests and Mann Whitney tests) as appropriate, using IBM® SPSS® Statistics version 22. In order to evaluate the quality of predictors associated with either latrine or marking problem, the sensitivity and specificity of the most significant predictors were calculated.

## Results

In total, 252 questionnaires were submitted with data; 245 were included in the analysis.

### Demographics

Overall, the countries with most respondents were in descending order Brazil (78, 31.8%), UK (47, 19.2%), Portugal (22, 9.0%), USA (19, 7.8%) and Australia (11, 4.5%) with 20 (8.2%) from other countries and 48 (19.6%) unknown. As expected the country of residence of respondents differed substantially between the two questionnaires, indicating that, as we had hoped, they were attracting different owner demographics which add to the diversity of the population sample (Data Sheet S2; Table S1).

The age of the cats ranged from 6 months to 19 years old (mean = 6.3 years, SD = 4.8) with 2 (0.8%) answers missing. Approximately half of the cats were male (125, 51.0%), with the vast majority having been neutered (229, 93.5%) and from non-specific breeds (208, 84.9%; Data Sheet S2; Table S2).

There was no significant difference between the two questionnaires (Brazilian and English) in the cats’ reproductive status, gender or pedigree status (Data Sheet S2; Table S3). The age range of cats in the two surveys was also similar (up to 17 years and 6 months for the Brazilian survey and 18 years and 4 months for the English survey), but there was a significant difference (*p* < 0.001) in the average age of cats (Brazil median 4 years, English median 7 years).

Approximately half (113, 46.1%) of cats included in the survey were reported to have never exhibited periuria. Of the remaining 132 cats, 40 (30.3%) were evaluated as exhibiting marking behaviour and 92 (69.7%) a latrine related problem; one cat was determined to be exhibiting both behaviours. Respondents of the Brazilian Portuguese survey had significantly (*p* < 0.05) fewer cats with no periuria (36.8% c.f. 54.7%) and a significantly higher proportion of individuals with latrine behaviour (49.6% c.f. 26.2%) even though observed marking was similar (13.7% c.f. 18.8% *p* > 0.05) between surveys (Data Sheet S2; Table S4).

### Individual Demographics

Neither gender, neuter status nor pedigree status  (Data Sheet S2; Table S5) were associated with the risk of either marking or latrine behaviour when compared to controls, but cats with a history of marking were significantly older (Data Sheet S2; Table S6) on average (median 9.5 years) than cats showing latrine behaviour (5 years) or no periuria (4 years) with *p* < 0.001.

### Household Features

The presence of at least one other cat in the house was associated with a higher frequency of periuria in general (OR = 3.006; CI_95%_ = 1.779–5.078, *p* < 0.001), with a six-fold increase in the risk of marking (OR = 6.384; CI_95%_ = 2.604–15.652; *p* < 0.001) and two-fold increase in latrine behaviour (OR = 2.310; CI_95%_ = 1.314–4.061; *p* = 0.003). Even though the number of cats in the household was significantly different between the groups (*p* < 0.001, Kruskal-Wallis), post hoc Mann Whitney test pairwise comparisons were not significant; nonetheless the median in the group without periuria was 1, whereas it was 2 in both the latrine and marking group.

The presence of cat flaps in the house (*p* = 0.012) and free outside access (*p* = 0.013) were associated with a higher prevalence of marking behaviour: OR_catflap_ = 2.730, CI_95%=_1.224–6.091 and OR_freeaccess_ = 2.859, CI_95%=_1.225–6.672); but on the other hand, outside access in general compared to no outside access was associated (*p* = 0.013) with a lower frequency of latrine behaviour in the home (OR = 0.486, CI_95%_ = 0.273–0.865). Neither the observed presence of neighbourhood cats near the house, nor the presence of another animal species in the home, including specifically a dog, was associated with an increased risk of periuria or any specific form of this (Data Sheet S2; Table S7). Data relating to significant household factors are summarized in Table 4.

**Table 4:** Significant potential household risk factors (Chi square test) for marking and latrine behaviour identified from the survey using univariate analysis.

Potential risk factor	Behaviour			Significance (2-sided)	Test stastical value
	Control (%) n	Marking (%) n	Latrine (%) n		
**Presence of another cat in the house**				*p* < 0.001	21.667
Yes	42.5% (48a)	82.5% (33b)	63.0% (58b)		
No	57.5% (65a)	17.5% (7b)	37.0% (34b)		
Missing	0.0% (0)	0.0% (0)	0.0% (0)		
**Cat flap in the house**				*p* = 0.005	10.591
Yes	17.7% (20b)	37.5% (15a)	13.0% (12b)		
No	80.5% (91b)	62.5% (25a)	84.8% (78b)		
Missing	1.8% (2)	0.0% (0)	2.2% (2)		
**Outside access allowed**				*p* = 0.003	16.411
Free access	14.2% (16b)	32.5% (13a)	12.0% (11b)		
Restricted access	33.6% (38a)	27.5% (11a)	19.6% (18a)		
No access	50.4% (57a)	40.0% (16a)	68.5% (63b)		
Missing	1.8% (2)	0.0% (0)	0.0% (0)		

Behavioural presentations (control, marking and latrine) differing at the 0.05 level of significance in post hoc pairwise comparisons are indicated by differing lower case letters, e.g., a vs b, of the same row, while those not differing (p > 0.05) have the same letter.

### Litter Box Attributes

No potential risk factors relating to the litter box which were investigated were found to be significantly associated with the risk of any form of periuria or periuria in general (Data Sheet S2; Table S8, S9). This included: the location of the litter box (divided into bedroom/living room vs laundry/kitchen vs hall/corridor vs bathroom vs other), whether the litterbox was cleaned of faeces or urine more than once daily or not, whether it was completely cleaned at least once a week or not, the use of a cleaner other than water to clean the box, the use of litter liners, the use of scented litter, the use of clumping litter, the use of fine grained, course or crystal litter, whether the box is open or covered, the size of the box (classified as small – one cat length, medium or large – more than two cat lengths), the number of litter boxes relative to the number of cats in the home (less than, equal or more than, and as a numerical proportion).

### Cat's Individual Behavioural and Medical Characteristics

By contrast several characteristics associated with the cat’s behaviour but not medical history were associated with specific forms of periuria (Table 5; Data Sheet S2; Table S10). The odds of urinary latrine behaviour in the home were 5 times higher when the cat was noted to have also defecated outside the litter box (CI_95%_ = 2.541–9.725, *p* < 0.001). In addition, cats who were described as being very heavily dependent on their owner/clingy as opposed to simply affectionate were less likely to exhibit latrine related behaviour in the home (OR = 0.124, CI95% = 0.042–0.368, *p* < 0.001). A tendency to describe the cat as having a relaxed personality was associated with a lower risk of marking behaviour (OR = 0.317, CI_95%_ = 0.144–0.696, *p* = 0.003). Being described by the owner as very demanding for attention, nervous, easily frustrated, moody, or aggressive (including specific aggressive behaviour to either humans or other cats) were not associated with an increased risk of periuria. Neither covering of urine in the litter box, nor a known history of past medical conditions (including specifically urinary tract disease), nor exhibiting typically masculine behaviours, nor the frequency of scratching or rubbing behaviour and their associated target substrates was associated with any change in the risk of any form of periuria.

**Table 5:** Significant potential risk factors (Chi square test) for marking and latrine related to cat's behavioural and medical characteristics.

**Potential risk factor**	**Behaviour**			**Significance (2-sided)**	**Test statistic value**
	**Control n (%)**	Marking n (%)	Latrine n (%)		
**Defecation in the home sometimes**				*p* < 0.001	23.939
Yes	14.2% (16a)	27.5% (11a,b)	44.6% (41b)		
No	85.8% (97a)	72.5% (29a,b)	54.3% (50b)		
Missing	0.0% (0)	0.0% (0)	1.1% (1)		
**Cat's bond with owner**				*p* < 0.001	18.050
Very heavily dependent	25.6% (29a)	20.0% (8a)	4.3% (4b)		
Affectionate bond	70.0% (79a)	80.0% (32a)	95.7% (88b)		
Missing	4.4% (5)	0.0% (0)	0.0% (0)		
**Relaxed cat**				*p* = 0.009	9.535
Yes	71.7% (81b)	52.5% (21a)	60.9% (56a,b)		
No	19.5% (22b)	45.0% (18a)	33.7% (31a,b)		
Missing	8.8% (10)	2.5% (1)	5.4% (5)		

Behavioural presentations (control, marking and latrine) differing at the 0.05 level of significance in post hoc pairwise comparisons are indicated by differing lower case letters, e.g., a vs b, of the same row, while those not differing (p > 0.05) have the same letter.

### Predictors Frequently Used to Differentiate Marking From Latrine Behaviour

Specific items answered only by owners of cats with a history of periuria (132 in total), relating to commonly reported predictors of either marking or latrine behaviour, were tested to verify their congruence with the final diagnosis. The odds of a diagnosis of marking were much higher for those cats described as standing to urinate (OR = 403.2, CI_95%_ = 45.08–3606.13, *p* < 0.001), those selecting a vertical surface (84.00, CI_95%_ = 17.05–413.80, *p* < 0.001) and not behaving as if trying to cover the soiled area (OR = 31.5, CI_95%_ = 9.63–103.08, *p* < 0.001), compared to those adopting a squatting posture, using a horizontal surface or appearing to make attempts to cover the area, respectively. A marking diagnosis was also around 5 times (CI_95%_ = 2.23–11.95, *p* < 0.001) more likely if either a small (a few drops) or medium (a few milliliters) volume of urine had been deposited rather than a large volume (emptying bladder). These four predictors were therefore examined for their sensitivity and specificity (Table 6).

**Table 6:** Sensitivity and specificity of the most significant predictors for urinary marking and latrine behaviour.

	**Predictor**			
	**Classic Posture**(standing vs squatting)	**Covering action**(present vs absent)	**Surface chosen**(horizontal vs vertical)	**Volume of urine**(small/medium vs large)
**Marking**				
**Sensitivity**	84.8%	88.2%	66.7%	74.4%
**Specificity**	98.6%	83.9%	97.7%	73.6%
**Latrine**				
**Sensitivity**	98.6%	80.8%	97.7%	64.0%
**Specificity**	86.8%	89.5%	75.0%	79.6%

Besides the significant predictors described above (Table 7), oestrus, in females (Table 7), was significantly more frequent (*p* = 0.001) with marking (4, 25.0%) than latrine (0, 0.0%) behaviour, though the number of intact females in our sample (*n* = 4, 3.5%) was very low compared to those neutered (*n* = 110, 96.5%). The rooms soiled by the cat, the tendency to deposit urine on a specific object, the frequency of soiling, the identifiable onset of a specific change or individual around the time of onset of periuria, the occurrence of a specific event immediately before an event, the occasional restriction of the cat from its normal place of elimination, the use of punishment to correct the problem, and the tendency to use something to treat the problem, the age of the cat at the onset of periuria and a general cessation in the use of the litterbox were not useful for differentiating marking from latrine behaviour (Data Sheet S2; Table S11, S12).

**Table 7:** Significant predictors of either marking or latrine behaviour (Chi square test).

Predictor	Behaviour		Significance (2-sided)	Test statistic value
	Marking n (%)	Latrine n (%)		
**Posture for house soiling**			*p* < 0.001	79.690
Standing	70.0% (28a)	1.1% (1b)		
Squatting	12.5% (5a)	78.2% (72b)		
Missing	17.5% (7)	20.7% (19)		
**Cat's behaviour after soiling**			*p* < 0.001	46.909
Does not act as if covering the area	75.0% (30a)	16.3% (15b)		
As if covering the area	10.0% (4a)	68.5% (63b)		
Missing	15.0% (6)	15.2% (14)		
**Surface for house soiling**			*p* < 0.001	59.911
Vertical surface	50.0% (20a)	2.2% (2b)		
Horizontal	25.0% (10a)	91.3% (84b)		
Missing	25.0% (10)	6.5% (6)		
**Amount of urine**			*p* < 0.001	16.033
A few drops ora few millilitres	72.5% (29a)	34.8% (32b)		
Emptying of the bladder	25.0% (10a)	62.0% (57b)		
Missing	2.5% (1)	3.3% (3)		
**For females only, if the behaviour is more frequent on oestrus**			*p* = 0.001	10.807
Yes	25.0% (4a)	0.0% (0b)		
No	56.3% (9a)	76.2% (32b)		
Missing	18.7% (3)	23.8% (10)		

The sum of individuals is less than 132 for the item about females in oestrus because only this gender was considered. Behavioural presentations (control, marking and latrine) differing at the 0.05 level of significance in post hoc pairwise comparisons are indicated by differing lower case letters, e.g., a vs b, of the same row, while those not differing (*p* > 0.05) have the same letter.

## Discussion

These results not only reinforce the evidence base for certain associations previously reported, but also shed new light on the strength of these and other new associations. By considering the breadth of factors discussed in the clinical behaviour literature, the results highlight the particular importance of the social (as opposed to physical) environment as general risk factors. It has been suggested previously that living in multi-cat households could be linked to urinary house soiling in cats (1, 18), but, prior to our study, statistical data only supported a risk in relation to marking behaviour, and this was weak (17). The presence of more than one cat in the house (but not the absolute number beyond two) was associated with periuria in our study. This indicates that the transition from solitary living to living with others (rather than the number of others) appears to be one of the main risk factors for urinary house soiling by many cats. Future work may be able to explore this relationship further and differentiate the risk associated with number of cats versus number of social groups in the home. We found that periuria was three times (95%CI = 1.779–5.078) more likely in multi-cat households, but the effect seems to be much greater on marking than latrine related behaviour (6-fold versus 2-fold increase in risk). It is interesting to note that in our study, 82.5% of the marking cats and 63.0% of the latrine ones lived in multi-cat households, which is remarkably similar to the values reported by Olm and Houpt (1) (84.0 and 74.0% respectively) 30 years ago in a US population, likewise Pryor et al., (17) found that 89% of marking cats lived in multicat households. These figures seem remarkably consistent, despite changes in cat ownership demographics over this time and advances in treatment in this time, such as the use of pheromone products (19). It is possible that the presence of one or more additional cats, not only disrupts social relationships but also interferes with access to physical resources in the environment, increasing the risk of competition over resources and access around the home, which might lead to both increased marking and latrine behaviour. This risk of occurrence may not have changed over time, as the risk factors are perhaps intrinsic to cats, and newer treatments, such as the use of pheromone products, seem to primarily reduce the frequency of signs such as spraying rather than producing an absolute cessation (44); thus it might be that the severity of the problem has declined but not its prevalence.

Previous authorities (e.g., 16) have indicated that defecation outside of the litter box is more likely among cats with either urinary marking or latrine related behaviour in the home, however, we found no data to support this general association; instead we found an association only with urinary latrine behaviour, with risk around 5 times higher (CI 95% = 2.541–9.725) in this case. This is consistent with the common triggers for urinary latrine behaviour in the home (such as the diverse litter box related factors which might vary between individual cases discussed below), being general enough to effect all elimination behaviour (i.e., the use of the litter box for defecation as well). By comparison, the link between urinary marking and middening (marking with faeces) may not be so strong, and the two may serve different functions, with middening generally being much less common than urinary marking (4). This makes it unlikely that we would detect a statistical association in a relatively small sample such as ours, which will only tease out the strongest effects, even though the prevalence of faeces outside the box was twice as common among urinary marking cats compared to their controls (Table 5). This factor therefore deserves investigation in a larger population, and it is worth noting that the current study cannot exclude certain factors as risk factors; it can only reveal the stronger associations.

Anecdotally, negative mental states, such as those induced by prolonged or frequent stressors, have been associated with house soiling issues (18, 27), and we found that cats described as having a relaxed personality were about a third (OR = 0.317) as likely to engage in marking within the home, but found no relationship with other personality factors or mood tendencies, such as nervousness (which has been suggested by other authors e.g., (22). Likewise, there seemed to be no association between the broad traits and tendencies we investigated and the risk of latrine behaviour in the home. Somewhat to our surprise, we also found no relationship, between known medical history and periuria or any form of this. It is widely believed that conditions like interstitial cystitis may affect the urgency of urination, volume eliminated and frequency of elimination leading to periuria in cats (16, 29). Our finding is consistent with the result of Tynes et al. (38) but not Frank et al. (37) who both examined the relationship between medical factors and spray marking; the lack of a general association with periuria is also in contrast to the report of Horwitz (2). However, until larger or better controlled studies, e.g., such as those involving comparisons between urinary house soiling and non-urinary house soiling cats drawn from the same home, are published, it is unlikely that we will be able to establish with any certainty what the relationship between medical problems and periuria in cats may be. Nonetheless it is worth noting from a clinical standpoint that there was a history of medical issues in at least 28.0% of cases in any group (control 28.3%, marking 37.5%, latrine 32.6%), and that the lower figure was in the control group. When the focus is just on urinary conditions, again the difference is not significant but the proportion showing latrine related problems (15.2%) was numerically higher than within the control or marking group which were both around 12.5%. Accordingly, we suggest it should remain standard practice to screen all cats presenting with periuria problems for medical issues, but it should not be assumed that if a health related problem is found that this is necessarily the cause, given the high prevalence of these issues in the population at large. Owners should be advised accordingly.

The lack of statistical association between any form of periuria and gender was another surprising result, However, previous studies which have identified a statistical association with marking (1, 17, 34) have been based on case series involving populations selected for one or more characteristics (e.g., one of the inclusion criteria for (17) was that subjects should be depositing four or more urine marks indoors on vertical surfaces per week); they have also been based in the US, and this may mean that there are previously unrecognized confounds affecting the results, such as the severity (e.g., number of marks made per day) of the condition, which might be greater in males and so result in them being probably more often referred for treatment. This hypothesis deserves further investigation. Neutering is widely believed to have a protective or therapeutic effect on marking behaviour (5, 17, 35), and while no effect of neutering was found in the current study, this is probably due to vast majority of cats (93.5%) in the current study being neutered.

The finding that the average age of cats with marking issues were significantly older on average (median = 9.5 years) than both cats in the latrine (5.0 years) and control (4.0 years) groups, but that the age of onset of marking and latrine behaviour is not significantly different (median = 2.0 and 1.5 years, respectively), is perhaps harder to explain. It might be that marking is better tolerated by owners, perhaps because it is less severe (less frequent, smaller volumes on average) or that owners are more aware of the association between this and environmental threats, so more willing to accept it as “normal” (whereas they may expect it to be normal for cats to use a litter box in the home), or related to distress. Either of these might result in the owner being less willing to rehome the cat or have it euthanized. Interestingly it was found that there was a significant difference in the owner-perceived bond of the cat to them depending on periuria, with a perceived “strong dependence on its owner” less common among cats in the latrine group (4.3%) compared to the other two groups (20–25%), and an “affectionate bond” more common (95.7%) in this group compared to the others (70–80%). It is possible that heavy dependence is viewed by many owners as a negative trait, and if this is combined with problem latrine behaviour in the home, it might be that this makes it much more likely that the cat is ejected from the home, by contrast over-demonstrations of affection might have a protective effect against this risk. This remains conjecture, but it would be useful to examine in more detail the relationship between owners’ attitudes to marking versus latrine related behaviour in the home, that cat’s personality or behavioural style and both the bond between owner and cat as well as the care the cat might receive if it has a urinary house soiling problem.

Around half of the cats in both the control (47.8%) and marking group (60%) were allowed some form of outside access, but less than a third (31.6%) of cats in the latrine group. This difference was significant, but completely free access (and the use of a cat flap) was associated with an increased risk of marking (Table 4). In this regard it is worth noting that 49% of owners of marking cats in the study of Pryor et al. (17) believed that agonistic interactions with other cats outside the house was the cause of the marking behaviour. Some veterinarians may also recommend restriction of outside access due to the increased risk of certain infectious diseases (such as FIV, FeLV and sporotrichosis), and accidental injuries or predation (45); but these risks clearly need to be balanced against the increased risk of latrine problems that appears to arise from being an indoor only cat. It is sometimes suggested that free access, through an uncontrolled cat flap may also allow neighbourhood cats to enter the property and threaten resources, which could trigger marking behaviour, but our results are not consistent with this suggestion, as there was not much difference between marking and controls in this feature; rather the risk seems to relate primarily to latrine behaviour. It is suggested that such risks can be mitigated by the use of a microchip controlled cat flap (46, 47), but there are currently no scientific data to support this. Allowing some access to the outside might increase opportunities to eliminate in general (via access to outside latrines) and thus reduce the risk that litter boxes in the home become unacceptable to the cat, perhaps because of the presence of faeces in it (20), as they are being used less frequently.

Several authors (1, 25, 32) have suggested a range of litter box attributes which may be important risk factors for latrine behaviour in cats. However, we found none of those that we investigated (e.g., size of litter box, absolute and relative number of litter boxes, type of litter, etc.) to be statistically associated with any form of periuria. These results do not mean that the litter environment is not important, only that there do not appear to be common factors across a substantial proportion of the population of cats with this problem. Although it is often recommended that the number of litter boxes in the home should equal the number of cats plus one, to prevent house soiling (18), we found that this rule of thumb is not followed by at least 70% of the cats in each subpopulation studied, including the control (control 70.8%, marking 70.0%, latrine 75.0%). These results are consistent with the suggestion that there are several common causes of latrine related problems, with different factors being significant in different cases. An effect of specific litter box factors might then only be revealed in a much larger survey. In the case of the number of litter trays in the two conditions described above, we calculate that to have an 80% chance of picking up a difference between the two populations at (*p* < 0.05) with this level of prevalence, we would need 1249 subjects in both the control and latrine group (https://www.sealedenvelope.com/power/binary-superiority/). Such a large sample may be difficult to achieve, but a larger sample than the current one, may be more amenable to multivariate analysis which may not only confirm the significance of the factors identified here, but also identify the best predictors of periuria in general, latrine related behaviour and marking in the home. This was not however the aim of the current study.

Very few studies have tried to quantify the potential value of particular signs in veterinary behavioural medicine (48) and there is a risk of potential circular reasoning, in so much as the signs being tested are those used to establish the diagnosis, which is a challenge in this type of work. In this instance, the diagnostic assessment was holistic with no specific algorithm followed (not least because a valid one does not exist), and the assessments were confirmed independently by a specialist used to dealing with atypical cases (DM) supported by others training towards specialism, since there is no objective gold standard available. By showing that the sensitivity and specificity of these signs is less than 100%, we not only demonstrate that individual signs are not being used for the diagnosis and thus the process cannot be entirely self-fulfilling, but also that individual signs are not reliable. However, we accept that the exact values may only relate to this population. Nonetheless, we believe the current study is the first to report specifically on sensitivity and specificity of predictors used to differentiate a common presenting complaint (in this case periuria). We specifically examined the quality of four features often used in the differential diagnosis of periuria (posture of cat during urinary soiling, perceived attempts at covering behaviour after the act, the orientation of the surface where urine was deposited and volume of urine). As expected all were significantly associated with the related form of periuria, however their reliability as either markers of the behaviour (sensitivity) or their absence as markers of the absence of the particular problem with which they are typically associated (specificity) was much more variable (Table 6). For example, if the production of a large volume of urine as opposed to a small to medium volume is used to determine that a cat is engaged in latrine behaviour then more than a third (36%) of cases with latrine related problems would have been missed and just over 20% (20.4%) of cats with marking problems misdiagnosed; likewise, if a small to medium volume of urine is used to determine that a cat is marking, around a quarter of marking cats would have been missed (25.6%) and a similar proportion (26.4%) of latrine cats misdiagnosed. Similarly, if the use of a vertical surface to deposit urine is used to determine that marking behaviour is occurring, then a third of cases (33.3%) would have been missed, but very few (2.3%) cats with latrine problems misdiagnosed; whereas the use of a horizontal surface for urine deposition will capture most cats with a latrine problem, it would have also included a quarter of cats with a marking issue. By contrast, posture for urine deposition and the presence or absence of apparent attempts to cover the urine, may be more reliable, there are still substantial issues with using these signs alone. These results highlight the importance of not using a single sign to determine diagnosis as well as the danger of simply gathering positive evidence in support of one particular diagnosis, rather than undertaking a comprehensive assessment of the case, focused on excluding the differentials.

We acknowledge a number of limitations to this study, which need to be appreciated for the results to be kept in context. Firstly, when convenience and snowball sampling are used, as in this case, only cat owners with internet access and social media involvement are able to answer the questionnaire, and this may not provide a representative population of owned cats. The analyses we have also undertaken are also rather simple and we have not attempted any multivariate modeling or examination of collinearity between the significant factors identified. Nonetheless, we argue that the approach reported here is the most appropriate analysis given the small sample size and the general lack data to date on this topic, so long as the relationships identified here are taken as tentative observations and kept in context. Such simple associations are nonetheless very valuable, since they are observations of what seems to be correlated, and provide a useful basis for initial hypothesis testing and reformulation. While multivariate analyses can account for interactions between these factors, they typically will reduce the data to the best explanatory variables and this might mean that clinically important relationships, (such as those that can be more effectively managed) may be lost in favour of better predictors. Thus the two different types of analyses are valuable in different ways. At this stage, given how little we know about the problem, we believe that providing evidence for the simple associations that might be observed is of most value. Later larger scale work will be able to model the best predictors (and is ongoing at present). Moreover, without a large sample, it is not recommended to focus on factor interactions, since it could mean the loss of individual important variables in favour of a small number of better predictors. These results question the soundness of many assumptions about the importance of several factors to the risk of periuria in cats and its two major forms (marking and latrine related behaviour). The differing risk factors for latrine related behaviour versus marking in the home reinforce the need to differentiate these conditions, and this must be done using a range of features concerning the presenting complaint, rather than single predictors, like posture, the volume of urine being deposited, the surface used or attempts to cover the urine afterwards. In particular, our findings highlight the importance of the amount and form of outdoor access as a risk factor in periuria, which affects these two conditions differently. Although we did not identify specific risk factors associated with the litter box that increased the risk of urinary latrine behaviour outside of the box, the association between this behaviour and defecation in the home, reinforces the belief that latrine related issues are often associated with some form of general aversion to the designated latrine area or some form of attraction to a new area. Our results have emphasized several areas for future research and, in particular the nature of the bond or perceived bond between the cat and its owner on both the risk of these problems and their management deserves further attention.

## Ethics Statement

This study and its protocol was covered by the ethical approval for the reporting of case material by the University of Lincoln Animal Behaviour Clinic, reference CoSREC281, granted by the College of Science Research Ethics Committee, University of Lincoln.

## Author Contributions

AB, DM initially designed and executed the study. AB, KM, NA, DM were all involved in the diagnosis of subjects and interpretation of results. AB, KM, NA, DM all contributed to the writing of this article.

## Conflict of Interest Statement

The authors declare that the research was conducted in the absence of any commercial or financial relationships that could be construed as a potential conflict of interest.
