# Supplementary material for: Common Risk Factors for Urinary House Soiling (Periuria) in Cats and Its Differentiation: The Sensitivity and Specificity of Common Diagnostic Signs
Source: Front Vet Sci. 2018 May 28;5:108. doi: 10.3389/fvets.2018.00108 (PMC5985598; doi:10.3389/fvets.2018.00108)
Supplement: Supplementary file 1 [file DataSheet1.DOCX]

**Appendix I**

**Questionnaire - English version**

1. **Please fill in the following information:**

Your country:

Your cat's name:

Your cat's age:

Your cat's gender:

Your cat's breed:

If your cat is neutered or not:

1. **Has your cat ever displayed urine marking or toileting behaviour in the home?**

Yes ☐ (Please, continue to answer the questionnaire)

No ☐ (Please, go to question 22)

1. **What is the cat’s position during the act? Please describe each form of the behaviour observed.**
2. **What is the cat’s behaviour after the act (e.g. does the cat sniff the area or behave as if covering the urine)? If this varies with the posture described in question 3 above, please indicate this clearly.**
3. **The urine marking/latrine behaviour is on:**

Vertical surfaces ☐

Horizontal surfaces ☐

Mostly vertical but also horizontal ☐

Mostly horizontal but also vertical ☐

Both equally ☐

1. **How much urine does the cat deposit outside the litter box?**

Small amount (small stains, a few drops) ☐

Medium amount (a few mls) ☐

Large amount (appears to empty its bladder) ☐

1. **Which is the room of the house most frequently used for urine marking/latrine behaviour? ---And in which room are the litter boxes? Please indicate the total number of other rooms in which the behaviour occurs and if they have any specific function, e.g. 2 bedrooms and kitchen.**
2. **Is the urine marking/latrine in a specific location?**

Yes ☐

No ☐ (go to question 10)

1. **What are these target objects/areas (in order of frequency used)? E.g. sofa, wall by the kitchen door, carpet, etc.**
2. **How often is the marking/latrine behaviour?**

Every time my cat urinates **☐**

A few times a day **☐**

Once a day **☐**

A few times a week **☐**

A few times a month **☐**

A few times a year **☐**

1. **When did the marking/latrine behaviour start? Please indicate the age of the cat at this time and how long ago this was.**
2. **Has anything changed in the cat’s environment? If yes, what? E.g: new child, new family member, new animal, new litter, new litter site.**
3. **Is there any special event that precedes the urine marking/toileting? E.g. visit at home.**

Yes ☐

No ☐

1. **What is the special event?**
2. **Do you suspect any cause for your cat's behaviour? What is it?**
3. **If it is a female cat, the marking/latrine behaviour occurs when she is in season (oestrus)? At a particular time of the year? Indicate months involved.**
4. **Is there anything which occasionally limits your cat from accessing its normal toileting area? If yes, what? E.g. other pet, closed door etc.**
5. **Your cat soils in the house with urine when:**

There are not people at home ☐

There are people at home ☐

Both situations ☐

1. **What do you do when you find a soiled area or when you see the cat soiling in the home?**
2. **Have you ever used any drug/product to treat your cat’s behaviour? If yes, what was used? Was it effective?**
3. **Your cat has completely stopped using its normal latrine to:**

Urinate ☐

Defecate ☐

Both ☐

None ☐

1. **How many cats do you have at home? What are their genders and neuter status?**
2. **How many litterboxes do you have and where are they located? If you do not have litter box in the home, then please go to question 35.**
3. **How often do you clean the litterbox completely (replace all litter and wash the litter tray)?**

Every day ☐

A few times a week ☐

Once a week ☐

Once a month ☐

A few times a year ☐

1. **How often do you just remove the faeces/urine, but leave most of the litter in place (perhaps topping it up if necessary)?**

Every time my cat urinates/defecates ☐

A few times a day ☐

Once a day ☐

A few times a week ☐

Once a week ☐

Between two and three times a month ☐

Once a month ☐

Less than once a month ☐

1. **What do you use to clean the litter box?**
2. **Do you use liners for your litter box?**

Yes ☐

No ☐

1. **After urinating in the litter box does the cat cover its urine?**

Yes ☐

No ☐

1. **After defecating in the litter box does the cat cover its feces?**

Yes ☐

No ☐

1. **While urinating in the litter box does the cat scratch outside the box such as the wall or the floor nearby?**

Yes ☐

No ☐

1. **While defecating in the litter box does the cat scratch outside the box such as the wall or the floor nearby?**

Yes ☐

No ☐

1. **While urinating in the litter box does the cat perch on the side or leave its front feet outside the box?**

Yes ☐

No ☐

1. **While defecating in the litter box does the cat perch on the side or leave its front feet outside the box?**

Yes ☐

No ☐

1. **Litter characteristics:**

Scented or not scented?

Clumping litter or not clumping litter?

Fine grains, course grains or crystals?

Covered or opened litter box?

Small (one cat body length), medium (larger than one cat body) or large (larger than two body lengths)?

1. **Does the cat also defecate in the home sometimes?**

Yes ☐

No ☐

1. **Does your cat have any previous history of medical problems? If yes, what and when?**
2. **Do you have a cat flap?**

Yes, operated by a microchip ☐

Yes, but not operated by a microchip ☐

No ☐

1. **Does your cat have access to the outside?**

Yes, free access ☐

Yes, restricted access ☐

No ☐

1. **Is there any restricted areas in the house to which the cat is not allowed to go (e.g. door kept shut)? If yes, which?**
2. **Are there neighbor cats coming into your garden, patio, roof etc? Where?**
3. **Does your cat have potentially masculine features such as odorous tom-cat urine, mounting behaviour or penile barbs? If so, please give details**
4. **Would you say your cat is very demanding for attention?**

Yes ☐

No ☐

1. **Would you say your cat is very demanding for other things it wants? If yes, for what (e.g water, food, to go outside)?**
2. **Which of the following best describes your cat's bond with you?**

No bond ☐

Affectionate bond but not overly dependent on you ☐

Very heavily dependent on you / clingy ☐

Don’t know ☐

1. **Would you describe your cat as a nervous character? If so, why?**
2. **Would you describe your cat as an easily frustrated character? If so, why?**
3. **Would you describe your cat as a moody individual? If so, why?**
4. **Would you describe your cat as a relaxed character? If so, why?**
5. **Is your cat aggressive? Tick all that apply**

Yes, to other cats of my own ☐

Yes, to neighbor cats ☐

Yes, to known people ☐

Yes, to unknown people ☐

No ☐

1. **Are there other animals free within the home (except cats)? If so, what species?**
2. **How much scratching your cat exhibit?**

Every day ☐

Several times a week ☐

Several times a month ☐

Rarely ☐

Almost never ☐

1. **What objects does your cat scratch?**
2. **How much chin and body rubbing does your cat exhibit on you, other family members or** familiar objects?

Very frequently ☐

Frequently ☐

Occasionally ☐

Rarely ☐

Almost never ☐

1. **How much chin and body rubbing does your cat exhibit on new people or new items in the home?**

Very frequently ☐

Frequently ☐

Occasionally ☐

Rarely ☐

Almost never ☐

1. **Does your cat exhibits pseudospraying (raising its tail and twitching it as if spraying but nothing producing any urine)? If so, in which situation?**
