# Supplementary material for: Common Risk Factors for Urinary House Soiling (Periuria) in Cats and Its Differentiation: The Sensitivity and Specificity of Common Diagnostic Signs
Source: Front Vet Sci. 2018 May 28;5:108. doi: 10.3389/fvets.2018.00108 (PMC5985598; doi:10.3389/fvets.2018.00108)
Supplement: Supplementary file 2 [file DataSheet2.DOCX]

***Supplementary material***

# Common Risk Factors for Urinary House Soiling (Periuria) in Cats and Its Differentiation: The Sensitivity and Specificity of Common Diagnostic Signs

**Ana Maria Barcelos, Kevin McPeake, Nadja Affenzeller and *Daniel Simon Mills**

***Correspondence:**

Prof. Daniel Simon Mills

dmills@lincoln.ac.uk

**Table S1** Owners' country for the Portuguese and English questionnaire

|  | **Portuguese**  **n (%)** | **English**  **n (%)** |
| --- | --- | --- |
| **Owners' country** |  |  |
| Brazil | 64_a_  (54.7%) | 14_b_  (10.9%) |
| UK | 0_a_  (0.0%) | 47_b_  (36.7%) |
| Portugal | 12_a_  (10.3%) | 10_a_  (7.8%) |
| USA | 1_a_  (0.8%) | 18_b_  (14.1%) |
| Australia | 0_a_  (0.0%) | 11_b_  (8.6%) |
| Others | 1_a_  (0.8%) | 19_b_  (14.8%) |
| Missing | 39  (33.3%) | 9  (7.0%) |

The percentage refers to the overall population of each Portuguese and English questionnaire.

**Table S2** Demographic characteristics of the cats included in the study

|  | **%** | **n** |
| --- | --- | --- |
| **Cats' gender** |  |  |
| Male | 51.0% | 125 |
| Female | 46.5% | 114 |
| Missing | 2.5% | 6 |
| **Cats' breed/type** |  |  |
| Domestic cat | 84.9% | 208 |
| Siamese | 4.9% | 12 |
| Persian | 3.7% | 9 |
| Bengal | 1.2% | 3 |
| Burmese | 1.2% | 3 |
| Maine Coon | 0.8% | 2 |
| Norwegian Forest cat | 0.8% | 2 |
| Ragdoll | 0.8% | 2 |
| Siberian | 0.4% | 1 |
| Missing | 1.2% | 3 |
| **Cats' reproductive status** |  |  |
| Neutered | 93.5% | 229 |
| Intact | 6.5% | 16 |
| Missing | 0.0% | 0 |

**Table S3** Reproductive status and gender of cats in the Portuguese and English questionnaire

|  | **Portuguese**  **n (%)** | **English**  **n (%)** | **Test** | **Significance**  **(2-sided)** |
| --- | --- | --- | --- | --- |
| **Cats' gender and reproductive status** |  |  | Chi-square | p=0.233 |
| Male neutered | 52_a_  (44.4%) | 61_a_  (47.7%) | z-test | 0.05 (level) |
| Male intact | 7_a_  (6.0%) | 5_a_  (3.9%) | z-test | 0.05 (level) |
| Female neutered | 53_a_  (45.3%) | 57_a_  (44.5%) | z-test | 0.05 (level) |
| Female intact | 3_a_  (2.6%) | 1_a_  (0.8%) | z-test | 0.05 (level) |
| Missing | 2  (1.7%) | 4  (3.1%) |  |  |
| **Breed** |  |  | Chi-square | 0.953 (2-sided) |
|  |  |  |  |  |
| No pedigree breed | 99_a_  (84.6%) | 109_a_  (85.2%) | z-test | 0.05 (level) |
| Pedigree breed | 16_a_  (13.7%) | 18_a_  (14.0%) | z-test | 0.05 (level) |
| Missing | 2  (1.7%) | 1  (0.8%) |  |  |

The percentage refers to the overall population of each Portuguese and English questionnaire.

**Table S4 D**istribution of periuria and its two forms in the Portuguese and English questionnaires

|  | **Portuguese**  **n (%)** | **English**  **n (%)** | **Test** | **Significance**  **(2-sided)** |
| --- | --- | --- | --- | --- |
| **Behaviour** |  |  | Chi-square | p=0.001 |
| No periuria | 43_a_  (36.8%) | 70_b_ (54.7%) | z-test | 0.05 (level) |
| Marking | 16_a_  (13.7%) | 24_a_  (18.8%) | z-test | 0.05 (level) |
| Latrine | 58_a_  (49.6%) | 34_b_  (26.6%) | z-test | 0.05 (level) |

The percentage refers to the overall population of each Portuguese and English questionnaire.

**Table S5** Non-significant categorical demographic risk factors for marking and latrine behaviour examined (missing was not used as a category in the analysis).

| **Potential risk factor** | **Behaviour** | | | **Significance**  **(2-sided)** | **df** | **Test** |
| --- | --- | --- | --- | --- | --- | --- |
|  | **Control**  n, std. residual | **Marking**  n, std. residual | **Latrine**  n, std. residual |  |  |  |
| **Gender** |  |  |  | p=0.601 | 2 | X^2^=1.018 |
| Male | 54 (47.8%)  -0.5 | 22 (55.0%)  0.5 | 49 (53.3%)  0.2 |  |  |  |
| Female | 56 (49.6%)  0.5 | 16 (40.0%)  -0.5 | 42 (45.7%)  -0.2 |  |  |  |
| Missing | 3 (2.7%) | 2 (5.0%) | 1 (1.1%) |  |  |  |
| **Reproductive status** |  |  |  | p=0.567 | 2 | X2=1.136 |
| Neutered | 107 (94.7%)  0.1 | 38 (95.0%)  0.1 | 84 (91.3%)  -0.2 |  |  |  |
| Intact | 6 (5.3%)  -0.5 | 2 (5.0%)  -0.4 | 8 (8.7%)  0.8 |  |  |  |
| Missing | 0 (0.0%) | 0 (0.0%) | 0 (0.0%) |  |  |  |
| **Pedigree** |  |  |  | p=0.269 | 2 | X2=2.624 |
| No pedigree breed | 94 (83.2%)  -0.1 | 32 (80.0%)  -0.4 | 82 (89.1%)  0.4 |  |  |  |
| Pedigree breed | 17 (15.0%)  0.4 | 8 (20.0%)  1.0 | 9 (9.8%)  -1.1 |  |  |  |
| Missing | 2(1.8%) | 0 (0.0%) | 1(1.1%) |  |  |  |

**Table S6** Association between age of cats and the risk of occurrence of marking and latrine behaviour

|  | **Behaviour** | | |  |  |
| --- | --- | --- | --- | --- | --- |
|  | **No periuria** | **Marking** | **Latrine** | **Test** | **Significance**  **(2-sided)** |
| **Age (in years) ***** |  |  |  | Kruskal-Wallis | p<0.001 |
| Median | 4a | 9.5b | 5a | Mann Whitney test | p<0.001 |
| Mean | 6.26 | 8.78 | 5.19 |  |  |
| 95% Confidence interval for mean | 5.37-7.15 | 7.22-10.33 | 4.25-6.13 |  |  |
| Std deviation | 4.76 | 4.86 | 4.52 |  |  |
| Range | 17.42 | 15.33 | 18.50 |  |  |
| Number of cats missing | 1 | 0 | 1 |  |  |

*** p<0.001. The subscript letters (a,b) are different among the columns when the potential risk factor differs among the behaviours at the 0.05 level of significance (asymptotic significance with 2-sided tests).

**Table S7** Potential household risk factors for marking and latrine behaviour

| **Potential risk factor** |  | **Behaviour** |  | **Significance (2-sided)** | **df** | **Test** |
| --- | --- | --- | --- | --- | --- | --- |
|  | **Control**  n, std. residual | **Marking**  n, std. residual | **Latrine**  n, std. residual |  |  |  |
| **Presence of another cat in the house ***** |  |  |  | p<0.001 | 2 | X2=21.667 |
| Yes | 48a (42.5%)  -2.0 | 33b (82.5%)  2.2 | 58b (63.0%)  0.8 |  |  |  |
| No | 65a (57.5%)  2.3 | 7b (17.5%)  -2.5 | 34b (37.0%)  -0.9 |  |  |  |
| Missing | 0 (0.0%) | 0 (0.0%) | 0 (0.0%) |  |  |  |
| **Cat flap in the house **** |  |  |  | p=0.005 | 2 | X2=10.591 |
| Yes | 20b (17.7%)  -0.4 | 15a (37.5%)  2.6 | 12b (13.0%)  -1.3 |  |  |  |
| No | 91b (80.5%)  0.2 | 25a (62.5%)  -1.3 | 78b (84.8%)  0.7 |  |  |  |
| Missing | 2 (1.8%) | 0 (0.0%) | 2 (2.2%) |  |  |  |
| **Outside access allowed **** |  |  |  | p=0.003 | 4 | X2=16.411 |
| Free access | 16b (14.2%)  -0.5 | 13a (32.5%)  2.5 | 11b (12.0%)  -1.1 |  |  |  |
| Restricted access | 38a (33.6%)  1.3 | 11a (27.5%)  0.0 | 18a (19.6%)  -1.5 |  |  |  |
| No access | 57a (50.4%)  -0.7 | 16a (40.0%)  -1.3 | 63b (68.5%)  1.6 |  |  |  |
| Missing | 2 (1.8%) | 0 (0.0%) | 0 (0.0%) |  |  |  |
| **Neighbour cats near the house** |  |  |  | p=0.125 | 2 | X2=4.160 |
| Yes | 46a (40.7%)  0.1 | 22a (55.0%)  1.2 | 33a (35.9%)  -0.9 |  |  |  |
| No | 58a (51.3%)  -0.1 | 17a (42.5%)  -1.1 | 56a (60.9%)  0.8 |  |  |  |
| Missing | 9 (8.0%) | 1 (2.5%) | 3 (3.3%) |  |  |  |
| **Another animal species at home** |  |  |  | p=0.422 | 2 | X2=1.728 |
| Yes | 48a (42.5%)  -0.7 | 22a (55.0%)  0.2 | 51a (55.4%)  0.6 |  |  |  |
| No | 53a (46.9%)  0.7 | 18a (45.0%)  -0.2 | 39a (42.4%)  -0.6 |  |  |  |
| Missing | 12 (10.6%) | 0 (0.0%) | 2 (2.2%) |  |  |  |
| **Dog at home** |  |  |  | p=0.387 | 2 | X2=1.899 |
| Yes | 44a (38.9%)  -0.7 | 21a (52.5%) 0.3 | 47a (51.1%)  0.6 |  |  |  |
| No | 57a (50.4%)  0.7 | 19a (47.5%)  -0.3 | 42a (45.7%)  -0.5 |  |  |  |
| Missing | 12 (10.6%) | 0 (0.0%) | 3 (3.3%) |  |  |  |

** = p<0.01; *** = p<0.001. The subscript letters (a,b) are different among the columns when the potential risk factor differs among the behaviours at the 0.05 level of the z-test.

**Table S8** Litter box attributes examined as potential risk factors for marking and latrine

| **Potential risk factor** | **Behaviour** | | | **Significance**  **(2-sided)** | **df** | **Test** |
| --- | --- | --- | --- | --- | --- | --- |
|  | **Control**  n, std. residual | **Marking**  n, std. residual | **Latrine**  n, std. residual |  |  |  |
| **Location of the litter box** |  |  |  | p=0.785 | 8 | X2=4.737 |
| Bedroom, living room | 17a (13.8%)  -0.8 | 11a (19.3%)  0.2 | 28a (22.6%)  0.6 |  |  |  |
| Laundry, kitchen | 30a (24.4%)  0.6 | 13a (22.8%)  -0.2 | 31a (25.0%)  -0.4 |  |  |  |
| Hall, corridor | 11a (8.9%)  0.2 | 8a (14.0%)  1.2 | 9a (7.3%)  -1.0 |  |  |  |
| Bathroom | 22a (17.9%)  -0.2 | 11a (19.3%)  -0.2 | 30a (24.2%)  0.3 |  |  |  |
| Other | 16a (13.0%)  0.2 | 6a (10.5%)  -0.7 | 20a (16.1%)  0.3 |  |  |  |
| Missing | 27 (22.0%) | 8 (14.0%) | 6 (4.8%) |  |  |  |
| **Litter box completely cleaned** |  |  |  | p=0.705 | 2 | X2=0.700 |
| At least once a week | 71a (62.8%)  -0.3 | 30a (75.0%) 0.2 | 70a (76.1%)  0.2 |  |  |  |
| Less than once a week | 25a (22.1%)  0.6 | 8a (20.0%)  -0.3 | 19a (20.7%)  -0.4 |  |  |  |
| Missing | 17 (15.0%) | 2 (5.0%) | 3 (3.3%) |  |  |  |
| **Faeces or urine removed from litter box** |  |  |  | p=0.096 | 2 | X2=4.684 |
| More than once a day | 44a (38.9%)  -0.8 | 17a (42.5%)  -0.5 | 54a (58.7%)  1.2 |  |  |  |
| Once a day or less | 52a (46.0%)  0.8 | 20a (50.0%)  0.5 | 35a (38.0%)  -1.2 |  |  |  |
| Missing | 17 (15.0%) | 3 (7.5%) | 3 (3.3%) |  |  |  |
| **Product used to clean litter box** |  |  |  | p=0.230 | 2 | X2=2.936 |
| Just water | 9a (8.0%)  -0.6 | 8a (20.0%)  1.4 | 10a (10.9%)  -0.4 |  |  |  |
| Cleaning product | 72a (63.7%)  0.2 | 28a (70.0%)  -0.6 | 74a (80.4%)  0.2 |  |  |  |
| Missing | 32 (28.3%) | 4 (10.0%) | 8 (8.7%) |  |  |  |
| **If liner is used on the litter box** |  |  |  | p=0.306 | 2 | X2=2.368 |
| Liner | 7a (6.2%)  -0.6 | 6a (15.0%)  1.3 | 8a (8.7%)  -0.2 |  |  |  |
| No liner | 86a (76.1%)  0.2 | 31a (77.5%)  -0.4 | 81a (88.0%)  0.1 |  |  |  |
| Missing | 20 (17.7%) | 3 (7.5%) | 3 (3.3%) |  |  |  |
| **If litter is scented** |  |  |  | p=0.182 | 2 | X2=3.412 |
| Scented | 18a (15.9%)  0.5 | 2a (5.0%)  -1.6 | 16a (17.4%)  0.4 |  |  |  |
| Not scented | 80a (70.8%)  -0.2 | 33a (82.5%)  0.7 | 72a (78.3%)  -0.2 |  |  |  |
| Missing | 15 (13.3%) | 5 (12.5%) | 4 (4.3%) |  |  |  |
| **If litter is clumping** |  |  |  | p=0.823 | 2 | X2=0.389 |
| Clumping | 64a (56.6%)  0.0 | 20a (50.0%)  -0.3 | 61a (66.3%)  0.2 |  |  |  |
| Not clumping | 32a (28.3%)  0.0 | 12a (30.0%)  0.4 | 28a (30.4%)  -0.3 |  |  |  |
| Missing | 17 (15.0%) | 8 (20.0%) | 3 (3.3%) |  |  |  |
| **Type of litter grain** |  |  |  | P=0.159 | 4 | X2=6.593 |
| Fine | 45a (39.8%)  0.8 | 14a (35.0%)  0.2 | 30a (32.6%)  -0.9 |  |  |  |
| Course | 34a (30.1%)  0.0 | 12a (30.0%)  0.2 | 30a (32.6%)  -0.1 |  |  |  |
| Crystal | 8a (7.1%)  -1.3 | 3a (7.5%)  -0.6 | 17a (18.5%)  1.7 |  |  |  |
| Missing | 26 (23.0%) | 11 (27.5%) | 15 (16.3%) |  |  |  |
| **If the litter box is open or covered** |  |  |  | p=0.127 | 4 | X2=7.175 |
| Open | 75a (66.4%)  0.3 | 24a (60.0%)  -0.7 | 67a (72.8%)  0.2 |  |  |  |
| Covered | 18a (15.9%)  0.6 | 6a (15.0%)  0.0 | 12a (13.0%)  -0.6 |  |  |  |
| Both | 6b (5.3%)  -1.4 | 8a (20.0%)  2.0 | 10a,b (10.9%)  0.2 |  |  |  |
| Missing | 14 (12.4%) | 2 (5.0%) | 3 (3.3%) |  |  |  |
| **Litter box size** |  |  |  | p=0.227 | 4 | X2=5.654 |
| Small | 13a (11.0%)  -0.8 | 5a (11.1%)  -0.7 | 21a (20.0%)  1.3 |  |  |  |
| Medium | 69a (58.5%)  0.7 | 24a (53.3%)  -0.5 | 59a (56.2%)  -0.4 |  |  |  |
| Large | 22a (18.6%)  -0.4 | 14a (31.1%)  1.3 | 21a (20.0%)  -0.5 |  |  |  |
| Missing | 14 (11.9%) | 2 (4.4%) | 4 (3.8%) |  |  |  |
|  |  |  |  |  |  |  |
| **Number of litter boxes in the house** |  |  |  | p=0.832 | 4 | X2=1.469 |
| Less than the  number of cats | 31a (27.4%)  -0.4 | 14a (35.0%)  0.5 | 30a (32.6%)  0.1 |  |  |  |
| Equal to the  number of cats | 49a (43.4%)  0.6 | 14a (35.0%)  -0.7 | 39a (42.4%)  -0.2 |  |  |  |
| More than the  number of cats | 20a (17.7%)  -0.4 | 9a (22.5%)  0.3 | 20a (21.7%)  0.2 |  |  |  |
| Missing | 13 (11.5%) | 3 (7.5%) | 3 (3.3%) |  |  |  |

The subscript letters (a,b) are different among the columns when the potential risk factor differs among the behaviours at the 0.05 level of the z-test.

**Table S9** Data relating to the proportion of litter boxes per number of cats in the house as a potential risk factor for periuria

|  | **Behaviour** | | |  |  |
| --- | --- | --- | --- | --- | --- |
|  | **No periuria** | **Marking** | **Latrine** | **Test** | **Significance**  **(2-sided)** |
| **Proportion of litter boxes per number of cats** |  |  |  | Kruskal-Wallis | p=0.855 |
| Median | 1.0 | 1.0 | 1.0 |  |  |
| Mean | 1.05 | 0.93 | 1.07 | Mann Whitney test | p=0.846 |
| 95% Confidence interval for mean | 0.91-1.19 | 0.78-1.08 | 0.94-1.21 |  |  |
| Std deviation | 0.69 | 0.46 | 0.64 |  |  |
| Range | 4.0 | 2.0 | 4.0 |  |  |
| Number of missing individuals | 1 | 0 | 1 |  |  |

**Table S10** Results of analysis of potential risk factors for periuria related to cats individual behaviour and medical characteristics

| **Potential risk factor** | **Behaviour** | | | **Significance**  **(2-sided)** | **df** | **Test** |
| --- | --- | --- | --- | --- | --- | --- |
|  | **Control**  n, std. residual | **Marking**  n, std. residual | **Latrine**  n, std. residual |  |  |  |
| **If the cat covers the urine deposited in the litter** |  |  |  | p=0.708 | 2 | X2=0.689 |
| Yes | 84a (74.3%)  0.2 | 29a (72.5%)  -0.2 | 71a (77.2%)  -0.1 |  |  |  |
| No | 16a (14.2%)  -0.5 | 8a (20.0%)  0.5 | 17a (18.5%)  0.2 |  |  |  |
| Missing | 13 (11.5%) | 3 (7.5%) | 4 (11.5%) |  |  |  |
| **Medical condition in the past** |  |  |  | p=0.535 | 2 | X2=1.251 |
| Yes | 32a (28.3%) | 15a (37.5%)  0.7 | 30a (32.6%)  0.2 |  |  |  |
| No | 81a (71.7%)  0.4 | 25a (62.5%)  -0.5 | 62a (67.4%)  -0.1 |  |  |  |
| Missing | 0 (0.0%) | 0 (0.0%) | 0 (0.0%) |  |  |  |
| **Urinary disease in the past** |  |  |  | p=0.824 | 2 | X2=0.387 |
| Yes | 14a (12.4%)  -0.3 | 5a (12.5%)  -0.2 | 14a (15.2%)  0.5 |  |  |  |
| No | 99a (87.6%)  0.1 | 35a (87.5%)  0.1 | 78a (84.8%)  -0.2 |  |  |  |
| Missing | 0 (0.0%) | 0 (0.0%) | 0 (0.0%) |  |  |  |
| **Defecation in the home sometimes***** |  |  |  | p<0.001 | 2 | X2=23.939 |
| Yes | 16a (14.2%)  -2.8 | 11a,b (27.5%)  0..0 | 41b (44.6%)  3.1 |  |  |  |
| No | 97a (85.8%)  1.7 | 29a,b (72.5%)  0.0 | 50b (54.3%)  -1.9 |  |  |  |
| Missing | 0 (0.0%) | 0 (0.0%) | 1 (1.1%) |  |  |  |
| **Masculine features** |  |  |  | p=0.092 | 2 | 4.772 |
| Yes | 6a (5.3%)  -1.5 | 7a (17.5%)  1.3 | 12a (13.0%)  0.7 |  |  |  |
| No | 88a (77.9%)  0.5 | 31a (77.5%)  -0.5 | 73a (79.4%)  -0.3 |  |  |  |
| Missing | 19 (16.8%) | 2 (5.0%) | 7 (7.6%) |  |  |  |
| **Very demanding for attention** |  |  |  | p=0.318 | 2 | X2=2.288 |
| Yes | 61a (54.0%)  0.2 | 24a (60.0%)  0.7 | 43a (46.7%)  -0.7 |  |  |  |
| No | 50a (44.2%)  -0.3 | 15a (37.5%)  -0.8 | 47a (51.1%)  0.8 |  |  |  |
| Missing | 2 (1.8%) | 1 (2.5%) | 2 (2.2%) |  |  |  |
| **Very demanding for what it wants** |  |  |  | p=0.870 | 2 | 0.278 |
| Yes | 78a (69.0%)  0.1 | 26a (65.0%)  -0.2 | 64a (69.6%)  0.0 |  |  |  |
| No | 29a (25.7%)  -0.2 | 12a (30.0%)  0.4 | 25a (27.2%)  0.0 |  |  |  |
| Missing | 6 (5.3%) | 2 (5.0%) | 3 (3.3%) |  |  |  |
| **Cat's bond with owner ***** |  |  |  | p<0.001 | 2 | X2=18.050 |
| Very heavily dependent | 29a (25.6%)  2.5 | 8a (20.0%)  0.4 | 4b (4.3%)  -3.0 |  |  |  |
| Affectionate bond | 79a (70.0%)  -1.1 | 32a (80.0%)  -0.2 | 88b (95.7%)  1.3 |  |  |  |
| Missing | 5 (4.4%) | 0 (0.0%) | 0 (0.0%) |  |  |  |
| **Nervous character** |  |  |  |  |  |  |
| Yes | 27a (23.9%)  -0.1 | 12a (30.0%)  0.6 | 22a (23.9%)  -0.3 | p=0.700 | 2 | X2=0.712 |
| No | 77a (68.1%)  0.0 | 26a (65.0%)  -0.4 | 68a (73.9%)  0.2 |  |  |  |
| Missing | 9 (8.0%) | 2 (5.0%) | 2 (2.2%) |  |  |  |
| **Easily frustrated** |  |  |  | p=0.798 | 2 | X2=0.452 |
| Yes | 15a (13.3%)  -0.4 | 6a (15.0%)  0.0 | 16a (17.4%)  0.5 |  |  |  |
| No | 89a (78.8%)  0.2 | 32a (80.0%)  0.0 | 73a (79.3%)  -0.2 |  |  |  |
| Missing | 9 (8.0%) | 2 (5.0%) | 3 (3.3%) |  |  |  |
| **Moody individual** |  |  |  | p=0.129 | 2 | X2=4.090 |
| Yes | 23a (20.4%)  -0.5 | 14a (35.0%)  1.6 | 18a (19.6%)  -0.6 |  |  |  |
| No | 85a (75.2%)  0.3 | 25a (62.5%)  1.6 | 70a (76.1%)  0.3 |  |  |  |
| Missing | 5 (4.4%) | 1 (2.5%) | 4 (4.3%) |  |  |  |
| **Relaxed cat**** |  |  |  | p=0.009 | 2 | X2=9.535 |
| Yes | 81b (71.7%)  1.2 | 21a (52.5%)  -1.1 | 56a,b (60.9%)  -0.5 |  |  |  |
| No | 22b (19.5%)  -1.8 | 18a (45.0%)  1.7 | 31a,b (33.7%)  0.8 |  |  |  |
| Missing | 10 (8.8%) | 1 (2.5%) | 5 (5.4%) |  |  |  |
| **Aggressive cat** |  |  |  | p=0.242 | 2 | X2=2.839 |
| Yes | 31a (27.4%)  -0.8 | 17a (42.5%)  1.1 | 31a (33.7%)  0.2 |  |  |  |
| No | 79a (69.9%)  0.6 | 23a (57.5%)  -0.7 | 60a (65.2%)  -0.1 |  |  |  |
| Missing | 3 (2.7%) | 0 (0.0%) | 1 (1.1%) |  |  |  |
| **Target of aggression** |  |  |  | p=0.348 | 4 | X2=4.455 |
| Another cat | 17a (15.0%)  -0.4 | 11a (27.5%)  0.1 | 21a (22.8%)  0.3 |  |  |  |
| Person | 7a (6.2%)  0.1 | 2a (5.0%)  -1.0 | 9a (9.8%)  0.6 |  |  |  |
| Both | 6a (5.3%)  0.7 | 4a (10.0%)  0.9 | 2a (2.2%)  -1.3 |  |  |  |
| Missing and not aggressive | 83 (73.5%) | 23 (57.5%) | 60 (65.2%) |  |  |  |
| **Frequency of scratching behaviour** |  |  |  | p=0.308 | 2 | X2=2.354 |
| At least once a day | 65a (57.5%)  0.8 | 20a (50.0%)  -0.3 | 45a (48.9%)  -0.6 |  |  |  |
| Less than once a day | 45a (39.8%)  -0.8 | 20a (50.0%)  0.3 | 47a (51.1%)  0.7 |  |  |  |
| Missing | 3 (2.7%) | 0 (0.0%) | 0 (0.0%) |  |  |  |
| **Target of scratching** |  |  |  | p=0.849 | 4 | X2=1.375 |
| Household object (e.g.sofa, carpet, table, chair) | 74a (49.3%)  -0.4 | 27a (50.0%)  -0.2 | 68a (56.2%)  0.6 |  |  |  |
| Cat scratcher | 65a (43.3%)  0.4 | 23a (42.6%)  0.1 | 47a (38.8%)  -0.5 |  |  |  |
| Outside (e.g. tree, wooden stump) | 9a (6.0%)  0.1 | 4a (7.4%)  0.5 | 6a (5.0%)  -0.4 |  |  |  |
| Missing | 2 (1.3%) | 0 (0.0%) | 0 (0.0%) |  |  |  |
| **Frequency of rubbing to familiar people** |  |  |  | p=0.987 | 4 | X2=0.334 |
| Frequently | 6a (5.3%)  -0.3 | 3a (7.5%)  0.3 | 6a (6.5%)  0.1 |  |  |  |
| Occasionally | 15a (13.3%)  0.2 | 5a (12.5%)  -0.1 | 11a (12.0%)  -0.2 |  |  |  |
| Rarely | 88a (77.9%)  0.0 | 32a (80.0%)  -0.1 | 74a (80.4%)  0.1 |  |  |  |
| Missing | 4 (3.5%) | 0 (0.0%) | 1 (1.1%) |  |  |  |
| **Frequency of rubbing to unfamiliar people** |  |  |  | p=0.899 | 4 | X2=1.069 |
| Rarely | 35a (31.0%)  -0.4 | 17a (42.5%)  0.4 | 28a (30.4%)  0.2 |  |  |  |
| Occasionally | 27a (23.9%)  0.4 | 8a (20.0%)  -0.7 | 18a (19.6%)  0.0 |  |  |  |
| Frequently | 33a (29.2%)  0.0 | 14a (35.0%)  0.2 | 23a (25.0%)  -0.2 |  |  |  |
| Missing | 18 (15.9%) | 1 (2.5%) | 23 (25.0%) |  |  |  |

** = p<0.01; *** = p<0.001. The subscript letters (a,b) are different among the columns when the potential risk factor differs among the behaviours at the 0.05 level of the z-test.

**Table S11** Predictors of either marking or latrine behaviour. The sum of individuals equals more than 132 for the predictor "Rooms soiled by the cat", since more than one option was allowed, and less than 132 on the item about female in oestrus because only this gender was considered.

| **Predictor** | **Behaviour** | | **Significance**  **(2-sided)** | **df** | **Test** |
| --- | --- | --- | --- | --- | --- |
|  | **Marking**  n, std. residual | **Latrine**  n, std. residual |  |  |  |
| **Posture for house soiling ***** |  |  | p<0.001 | 1 | X2=79.690 |
| Standing | 28a (70.0%)  6.3 | 1b (1.1%)  -4.2 |  |  |  |
| Squatting | 5a (12.5%)  -3.9 | 72b (78.2%)  2.6 |  |  |  |
| Missing | 7 (17.5%) | 19 (20.7%) |  |  |  |
| **Cat's behaviour after soiling ***** |  |  | p<0.001 | 1 | X2=46.909 |
| Does not act  as if covering the area | 30a (75.0%)  4.4 | 15b (16.3%)  -2.9 |  |  |  |
| As if covering the area | 4a (10.0%)  -3.6 | 63b (68.5%)  2.4 |  |  |  |
| Missing | 6 (15.0%) | 14 (15.2%) |  |  |  |
| **Surface for house soiling ***** |  |  | p<0.001 | 1 | X2=59.911 |
| Vertical surface | 20a (50.0%)  5.1 | 2b (2.2%)  -3.5 |  |  |  |
| Horizontal | 10 (25.0%)  -3.5 | 84b (91.3%)  1.7 |  |  |  |
| Missing | 10 (25.0%) | 6 (6.5%) |  |  |  |
| **Amount of urine ***** |  |  | p<0.001 | 1 | X2=16.033 |
| A few drops or  a few millilitres | 29a (72.5%)  2.4 | 32b (34.8%)  -1.6 |  |  |  |
| Emptying  of the bladder | 10a (25.0%)  -2.3 | 57b (62.0%)  1.5 |  |  |  |
| Missing | 1 (2.5%) | 3 (3.3%) |  |  |  |
| **Rooms soiled by the cat** |  |  | p=0.272 | 4 | X2=5.150 |
| Living room or bedroom | 25a (48.1%)  0.9 | 50a (42.4%)  -0.6 |  |  |  |
| Kitchen or laundry | 8a (15.4%)  -0.1 | 22a (18.6%)  0.1 |  |  |  |
| Hall or corridor | 6a (11.5%)  0.6 | 11a (9.3%)  -0.4 |  |  |  |
| Bathroom | 4a (7.7%)  -1.3 | 23a (19.5%)  0.8 |  |  |  |
| Others | 1a (1.9%)  -1.0 | 8a (6.8%)  0.6 |  |  |  |
| Missing | 8 (15.4%) | 4 (3.4%) |  |  |  |
| **If there is a specific object or location soiled by the cat** |  |  | p=0.739 | 1 | X2=0.111 |
| Yes | 26a (65.0%)  0.2 | 57a (62.0%)  -0.1 |  |  |  |
| No | 14a (35.0%)  -0.2 | 35a (38.0%)  0.1 |  |  |  |
| Missing | 0 (0.0%) | 0 (0.0%) |  |  |  |
| **Frequency of house soiling behaviour** |  |  | p=0.904 | 2 | X2=0.201 |
| More than once a week | 16a (40.0%)  0.2 | 36a (39.1%)  -0.1 |  |  |  |
| A few times a month | 9a (22.5%)  -0.3 | 25a (27.2%)  0.2 |  |  |  |
| A few times a year | 13a (32.5%)  0.1 | 30a (32.6%)  -0.1 |  |  |  |
| Missing | 2 (5.0%) | 1 (1.1%) |  |  |  |
| **Any change in the environment before the house soiling onset** |  |  | p=0.788 | 1 | X2=0.072 |
| Yes | 26a (65.0%)  -0.1 | 60a (65.2%)  0.1 |  |  |  |
| No | 14a (35.0%) | 29a (31.5%)  -0.1 |  |  |  |
| Missing | 0 (0.0%) | 3 (3.3%) |  |  |  |
| **Any special event that precedes the house soiling** |  |  | p=0.541 | 1 | X2=0.373 |
| Yes | 10a (25.0%)  0.4 | 19a (20.6%)  -0.3 |  |  |  |
| No | 28a (70.0%)  -0.2 | 70a (76.1%)  0.2 |  |  |  |
| Missing | 2 (5.0%) | 3 (3.3%) |  |  |  |
| **For females only, if the behaviour is more frequent on oestrus **** |  |  | p=0.001 | 1 | X2=10.807 |
| Yes | 4a (25.0%)  2.6 | 0b (0.0%)  -1.7 |  |  |  |
| No | 9a (56.3%)  -0.8 | 32b (76.2%)  0.5 |  |  |  |
| Missing | 3 (18.7%) | 10 (23.8%) |  |  |  |
| **Anything, occasionally, limits the cat from accessing toileting area** |  |  | p=0.230 | 1 | X2=1.443 |
| Yes | 4a (10.0%)  -0.9 | 16a (17.4%)  0.6 |  |  |  |
| No | 33a (82.5%)  0.4 | 65a (70.7%)  -0.3 |  |  |  |
| Missing | 3 (7.5%) | 11 (12.0%) |  |  |  |
| **When the house soiling occurs** |  |  | p=0.770 | 2 | X2=0.523 |
| With people or without people in the house | 31a (77.5%)  0.2 | 66a (71.7%)  -0.1 |  |  |  |
| With people in the house only | 5a (12.5%)  0.2 | 10a (10.9%)  -0.1 |  |  |  |
| Without people in the house only | 4a (10.0%)  -0.6 | 13a (14.1%)  0.4 |  |  |  |
| Missing | 0 (0.0%) | 3 (3.3%) |  |  |  |
| **If the periuria has been punished (verbally, physically, visually)** |  |  | p=0.871 | 1 | X2=0.026 |
| Yes | 12a (30.0%)  0.1 | 27a (29.3%)  -0.1 |  |  |  |
| No | 27a (67.5%)  -0.1 | 65a (70.7%) |  |  |  |
| Missing | 1 (2.5%) | 0 (0.0%) |  |  |  |
| **If any product or drug has been used to treat the house soiling** |  |  | p=0.205 | 1 | X2=1.609 |
| Yes | 18a (45.0%)  0.8 | 30a (32.6%)  -0.6 |  |  |  |
| No | 20a (50.0%)  -0.7 | 55a (59.8%)  0.4 |  |  |  |
| Missing | 2 (5.0%) | 7 (7.6%) |  |  |  |
| **If the cat has stopped using the litter box for either urinating or defecating** |  |  | p=0.466 | 2 | X2=1.528 |
| Yes | 2a (5.0%)  -1.0 | 11a (12.0%)  0.5 |  |  |  |
| No | 30a (75.0%)  0.4 | 65a (70.7%)  -0.2 |  |  |  |
| Missing | 8 (20.0%) | 16 (17.4%) |  |  |  |

** = p<0.01; *** = p<0.001. The subscript letters (a,b) are different among the columns when the potential risk factor differs among the behaviours at the 0.05 level of the z-test.

**Table S12** Age when house soiling with urine started

|  | **Behaviour** | |  | **Significance**  **(2-sided)** |
| --- | --- | --- | --- | --- |
|  | **Marking** | **Latrine** |  |  |
| **Age periuria started** |  |  | Kruskal-Wallis | p=0.688 |
|  | 2.0 | 1.5 |  |  |
|  | 3.07 | 2.91 | Mann Whitney | p=0.459 |
|  | 1.85-4.28 | 2.13-3.69 |  |  |
|  | 3.31 | 3.58 |  |  |
|  | 11.75 | 16.88 |  |  |
| Number of missing individuals | 9 | 8 |  |  |
